# Modulation of Stress and Anabolic Signalling Pathways by Whey Protein Isolate in C2C12 Cells Under Exercise-Mimetic Conditions

**DOI:** 10.3390/biomedicines14061330

**Published:** 2026-06-11

**Authors:** Simone Mulè, Rebecca Galla, Matteo Musu, Francesca Parini, Francesca Uberti

**Affiliations:** 1 Department for Sustainable Development and Ecological Transition, University of Piemonte Orientale Piazza Sant’Eusebio 5, 13100 Vercelli, Italy; simone.mule@uniupo.it (S.M.); 20049586@studenti.uniupo.it (M.M.); 2 Noivita S.r.l.s., Spin Off, University of Piemonte Orientale, Strada Privata Curti 7, 28100 Novara, Italy; rebecca.galla@noivita.it (R.G.); francescaparini00@gmail.com (F.P.)

**Keywords:** whey protein isolate, skeletal muscle, C2C12 cells, exercise-mimetic stress, cellular stress response, anabolic signalling, mTOR pathway, oxidative stress, bioenergetics, myokines

## Abstract

**Background/Objectives**: Skeletal muscle adaptation to metabolic stress involves a coordinated regulation of inflammatory, bioenergetic, and anabolic signalling pathways. This study aimed to investigate the potential role of whey protein isolate (WPI; commercial name: Volapure) as a modulator of cellular responses to stress in an in vitro model of exercise-mimetic stress over time. **Methods**: Murine C2C12-differentiated cells were exposed to an Exercise–Mimetic Mix (ExM) to reproduce key biochemical features of muscle stress. Cells were treated with WPI (1 mg/mL) using Pre-exposure (Pre-ExM) and Post-exposure (Post-ExM) protocols at 8 and 24 h. Multiple endpoints were assessed, including cell viability, reactive oxygen species (ROS) production, cytokine release (TNF-α, IL-6, IL-17), intracellular signalling pathways (p38 MAPK, ERK, AMPK, mTOR), bioenergetic markers (ATP, glycogen, lactate), protein synthesis (OPP incorporation), and Ca^2+^/Mg^2+^ fluxes. **Results**: ExM exposure induced a stress phenotype characterised by increased oxidative and inflammatory markers, impaired bioenergetic status, and reduced anabolic signalling. WPI was associated with modulation of these responses, reducing ROS and pro-inflammatory cytokines, restoring ATP and glycogen levels, and changes in ERK and mTOR-related signalling. The Post-ExM protocol showed greater modulation compared to the Pre-ExM approach, particularly at 24 h. WPI was also associated with the normalisation of ExM-altered Ca^2+^/Mg^2+^ fluxes. These findings should be interpreted as associative rather than causal. **Conclusions**: WPI was associated with modulation of key pathways involved in cellular adaptation to metabolic stress, supporting recovery of bioenergetic balance and anabolic signalling in C2C12 cells. These findings suggest a potential role for WPI in influencing cellular responses to metabolic stress, supporting recovery of bioenergetic balance and anabolic signalling in C2C12-differentiated-cells. However, further studies are required to confirm the translational relevance of these observations.

## 1. Introduction

Skeletal muscle, one of the largest tissues in the human body, functions not only as the main driver for locomotion and postural support but also as a central metabolic hub essential for systemic energy homeostasis [1]. Besides its mechanical role, skeletal muscle acts as a key site for glucose disposal and lipid oxidation, greatly contributing to whole-body energy balance [2]. Maintaining muscle mass and function depends on a delicate balance between protein synthesis and breakdown, regulated by physical activity, hormonal signals, and nutritional status [3].

At the cellular level, amino acids (AAs) act not only as substrates for protein synthesis but also as key regulators of intracellular signalling pathways involved in muscle growth and adaptation [4]. Branched-chain amino acids (BCAAs), particularly leucine, isoleucine, and valine, activate mammalian target of rapamycin complex 1 (mTORC1), promoting ribosomal biogenesis and mRNA translation [5], while also modulating the phosphoinositide 3-kinase/protein kinase B (PI3K/Akt) pathway to support cell survival and growth [6]. Conversely, AMP-activated protein kinase (AMPK) functions as a central energy sensor during metabolic stress, inhibiting anabolic pathways and emphasising the importance of a dynamic balance between mTOR and AMPK signalling for muscle homeostasis [7,8]. Additionally, AAs regulate myogenic commitment and differentiation by modulating myogenic regulatory factors (MRFs) [9].

Muscle activity is inherently linked to metabolic stress. During intense contraction, reactive oxygen species (ROS) are produced, contributing to acute fatigue and, when dysregulated, to cellular damage and impaired regeneration [10]. Likewise, chronic elevation of pro-inflammatory cytokines, such as tumour necrosis factor α (TNFα) and interleukin-1 (IL-1), damages muscle integrity and promotes catabolic processes [11]. Emerging evidence suggests that AAs can influence oxidative stress and inflammatory pathways, reducing protein breakdown via the ubiquitin–proteasome and autophagic pathways [12].

In this context, skeletal muscle is increasingly recognised as an endocrine organ capable of releasing myokines that mediate inter-organ communication [13]. Among these, irisin, a cleavage product of fibronectin type III domain-containing protein 5 (FNDC5), has been linked to the regulation of glucose metabolism, mitochondrial function, and oxidative stress responses [14,15]. However, its role and quantification in in vitro systems remain under active investigation.

To examine these mechanisms under controlled conditions, in vitro models serve as a valuable tool. Murine C2C12 myoblasts replicate key stages of myogenesis and enable the investigation of signalling pathways involved in muscle adaptation [16]. In particular, the p38 mitogen-activated protein kinase (MAPK) pathway acts as a molecular switch that controls the transition from proliferation to differentiation and influences mitochondrial biogenesis and metabolic adaptation [17,18,19].

Dietary proteins, especially whey protein isolate (WPI), are commonly used to support muscle metabolism and recovery due to their high essential amino acid content and rapid absorption [20,21]. Beyond their nutritional function, these protein sources may affect intracellular signalling pathways involved in proteostasis and stress response [22]. However, the extent to which WPI can influence integrated cellular responses to metabolic stress, rather than merely providing substrates for protein synthesis, remains only partially understood.

To address this gap, the present study was designed to investigate the role of WPI in modulating cellular adaptation to exercise-mimetic stress. C2C12-differentiated cells were exposed to an Exercise–Mimetic Mix (ExM), consisting of an AMPK activator, caffeine, ionomycin, and a protein kinase A activator, to reproduce key biochemical features of muscle stress in vitro, as previously described for pharmacological exercise-mimetic approaches targeting AMPK, Ca^2+^, and cAMP/PKA signaling pathways in skeletal muscle models [23]. Pre-exposure (Pre-ExM) and Post-exposure (Post-ExM) conditions with WPI treatment protocols were applied to investigate temporal differences in cellular responses to WPI stimulation relative to the ExM exposure. A comprehensive set of endpoints was evaluated, including oxidative stress, inflammatory mediators, bioenergetic status, intracellular signalling pathways, protein synthesis, and Ca^2+^/Mg^2+^ fluxes. In parallel, chemical characterisation of whey protein isolate, including structural analyses by Fourier transform Infrared (FT-IR) spectroscopy, was conducted to investigate potential modifications induced by the cellular environment and their possible impact on bioavailability and functional effects.

This integrated approach, combining simulated gastrointestinal digestion (INFOGEST), structural analysis (FT-IR), and time-dependent cellular modelling, enables investigation of the potential role of digestion-derived peptides and amino acids in modulating cellular responses within the experimental model.

## 2. Materials and Methods

### 2.1. Materials

WPI (commercial name: Volapure) was provided by Yamamoto S.p.A. (Milan, Italy). Dulbecco’s Modified Eagle’s Medium (DMEM), fetal bovine serum (FBS), penicillin–streptomycin, and L-glutamine were obtained from Merck Life Science (Milan, Italy). 5-Aminoimidazole-4-carboxamide ribonucleoside (AICAR), caffeine, and 8-Bromo-cAMP sodium salt were purchased from Merck Life Science (Milan, Italy), while ionomycin calcium salt was obtained from Cayman Chemical (Ann Arbor, MI, USA).

Commercial assay kits used in this study included 3-(4,5-dimethylthiazol-2-yl)-2,5-diphenyltetrazolium bromide (MTT) viability assay, Enzyme-linked immunosorbent assay (ELISA) kits for cytokines (TNF-α, IL-6, IL-17) and irisin, adenosine triphosphate (ATP) assay kit, glycogen assay kit, lactate assay kit, mTOR and phospho-AMPK ELISA kits, LDH release ELISA kit, and Global Protein Synthesis (OPP) assay kit, obtained from the respective manufacturers as specified in each assay section.

Fluorescent probes for calcium and magnesium measurements (Fura-2 AM and Mag-fura-2 AM) were obtained from Merck Life Science and Biotium (Fremont, CA, USA), respectively.

All reagents were of analytical grade and prepared according to the manufacturers’ instructions.

### 2.2. Agents Preparation

The sample under examination, referred to as WPI, is a whey protein powder isolate characterised by high analytical purity, with a protein content of 88%. The amino acid profile, expressed in % (g/100 g), includes L-glutamic acid (17.0%), L-aspartic acid (11.0%), L-lysine (9.3%), L-leucine (9.2%), L-threonine (6.7%), L-proline (5.9%), and L-isoleucine (5.1%), with all other amino acids present at less than 5%.

WPI was freshly prepared prior to each experiment. For dose–response considerations in C2C12-differentiated cells, concentrations ranging from 0.05 to 5 mg/mL were selected based on previous in vitro studies reporting biological effects of whey protein in skeletal muscle cell models [24,25]. Within this experimentally supported range, the concentration of 1 mg/mL was selected for the main experiments as an intermediate dose commonly used in the literature and compatible with maintaining cell viability under comparable experimental conditions in C2C12-differentiated cells [25].

WPI was diluted in phenol red-free DMEM supplemented with 0% FBS, 50 IU/mL penicillin–streptomycin, and 2 mM L-glutamine. Prior to cell exposure, WPI samples were subjected to a standardised in vitro static digestion protocol according to the international INFOGEST consensus, as described in Appendix A. The resulting digested WPI represents a complex and heterogeneous mixture of peptides and free amino acids, and its exact molecular composition was not further characterised in this study.

An exercise–mimetic chemical induction mixture (Exercise–Mimetic Mix, ExM) was prepared using the same medium and included AICAR, caffeine, ionomycin, and 8-Bromo-cAMP to reproduce key biochemical features of muscle contraction and metabolic stress [23]. Final concentrations were: AICAR 1 mM (0.26 mg/mL), caffeine 2 mM (0.39 mg/mL), ionomycin calcium salt 0.5 µM (0.37 µg/mL), and 8-Bromo-cAMP sodium salt 100 µM (0.043 mg/mL) [26,27,28,29,30]. These concentrations were selected to activate relevant signalling pathways while minimising cytotoxicity, as reported in analogous in vitro studies [26,27,28,29,30].

### 2.3. Cell Culture

C2C12 murine myoblasts (American Type Culture Collection, ATCC, Manassas, VA, USA) were cultured in DMEM supplemented with 10% FBS and 100 U/mL penicillin–streptomycin. Cells were maintained at 37 °C in a humidified atmosphere with 5% CO_2_ and used at 40–70% confluence to allow proliferation while minimising spontaneous differentiation [31]. C2C12 cells proliferate as mononucleated myoblasts and differentiate into multinucleated myotubes when cultured in appropriate differentiation medium. Prior to seeding, serum concentration was reduced to 2% for 7 days to induce myogenic differentiation. This model provides a reproducible and scalable platform for studying key aspects of myogenesis, gene expression regulation, metabolic responses, and muscle-specific signalling pathways. Furthermore, C2C12 cells have been extensively utilised for drug screening, disease modelling, and the investigation of muscle atrophy, hypertrophy, and regeneration [16,32]. Differentiated cells are hereafter referred to as C2C12-differentiated cells in the text.

Cells were seeded according to assay requirements: 1 × 10^4^ cells/well in 96-well plates for viability, ROS, LDH, cytokine, ATP, and ion flux measurements; and 1 × 10^5^ cells/well in 6-well plates for protein and metabolic analyses.

All assays were normalised to total protein content to reduce variability across experimental conditions.

### 2.4. Experimental Design

**Figure 1:**
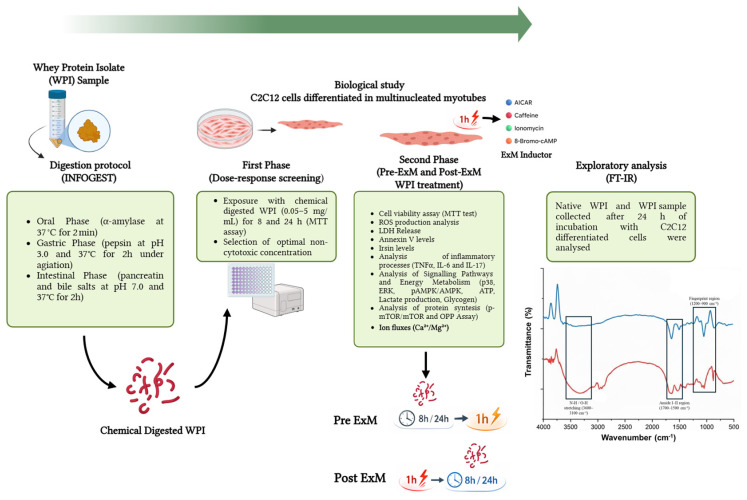
Schematic representation of the experimental workflow, including the INFOGEST digestion process, the two phases of biological analyses, and the exploratory FT-IR characterization study.

The experimental design consisted of two sequential biological phases: an initial dose–response screening aimed at identifying the optimal WPI concentration at the cellular level, followed by a second phase in which Pre-ExM and Post-ExM WPI treatment conditions were compared relative to the ExM stimulus. Furthermore, an exploratory phase with analysis of the structural changes of the WPI was conducted via Fourier transform infrared spectroscopy (FT-IR) and described later. Prior to the experiments, WPI samples were subjected to a standardised in vitro static digestion protocol following the international INFOGEST consensus, simulating the oral, gastric, and intestinal phases. This enzymatic pretreatment was applied to mimic gastrointestinal digestion and to obtain peptide fractions and free amino acids, which are generally considered the main bioaccessible components potentially reaching peripheral tissues under physiological conditions. The flowchart of the various experimental activities (INFOGEST; two phases of biological analyses and FT-IR study) is reported in Figure 1.

In the first phase, a dose–response analysis was performed using MTT assay following exposure to WPI (0.05–5 mg/mL) for 8 and 24 h. Based on these results, 1 mg/mL was selected as the optimal non-cytotoxic concentration.

In the second phase, C2C12-differentiated cells were exposed to ExM for 1 h to induce metabolic stress. The 1 h exposure duration was chosen to assess early signaling events associated with acute activation of exercise-responsive pathways, including AMPK-, Ca^2+^-, and cAMP/PKA-dependent signaling. This short-term stimulation is commonly employed in skeletal muscle cell models to evaluate rapid phosphorylation-mediated responses while limiting the contribution of downstream transcriptional or metabolic adaptations [23,33]. Two treatment protocols were applied: (i) Pre-ExM, consisting of WPI treatment (1 mg/mL) for 8 or 24 h prior to ExM exposure; samples were collected immediately after 1 h of ExM stimulation; and (ii) Post-ExM, consisting of ExM exposure for 1 h followed by WPI treatment (1 mg/mL), with samples collected at 8 h and 24 h after the stress stimulus.

Cells were harvested either immediately at the end of the 1 h ExM exposure (acute condition; black column in the graph in Section 3) or after the corresponding post-WPI treatment incubation periods (8 h and 24 h) in the Pre-ExM and Post-ExM protocols, without any additional recovery period, to assess phosphorylation-dependent signaling events associated with pathway activation. No visible contractile activity was observed in C2C12-differentiated following ExM exposure, consistent with the chemical nature of the stimulation, which does not involve electrical or neuromuscular activation. Following treatments, multiple endpoints were assessed, including oxidative stress, inflammatory cytokines (TNF-α, IL-6, IL-17), myokine secretion (irisin), intracellular signalling pathways (p38 MAPK, ERK, AMPK), bioenergetic parameters (ATP, glycogen, lactate), protein synthesis, and Ca^2+^/Mg^2+^ fluxes.

An exploratory analysis focused on studying the molecular-level structural changes of WPI induced by cellular interaction; Fourier transform infrared spectroscopy (FT-IR) was performed. Native WPI sample and WPI sample collected after 24 h of incubation with C2C12-differentiated cells were analysed to evaluate alterations in protein secondary structure and hydrogen-bonding patterns. Spectral regions corresponding to amide bands I and II, as well as N–H/O–H stretching, were examined to detect conformational remodelling associated with metabolic processing of WPI in the muscle cell environment.

**Figure A1:**
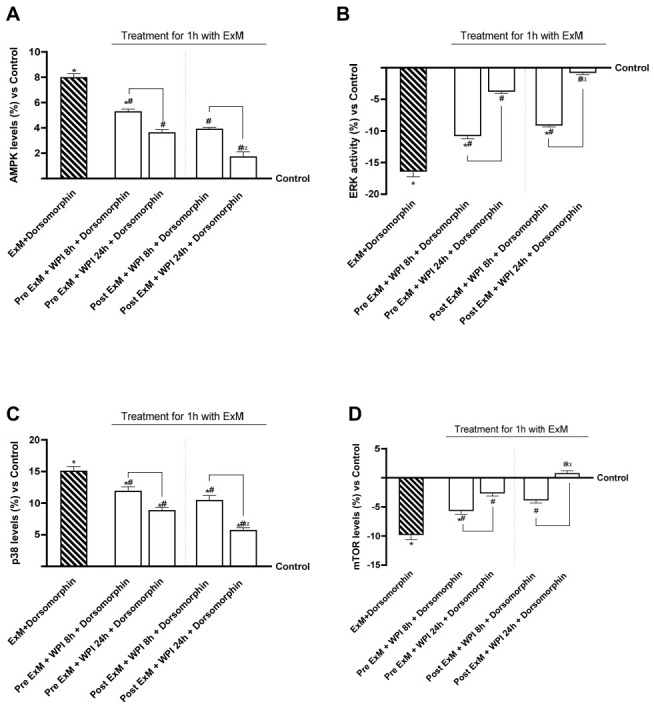
Exploratory analysis of pharmacological AMPK inhibition on AMPK, ERK, p38 MAPK, and mTOR signalling pathways in C2C12-differentiated cells under ExM-induced metabolic stress conditions. Signalling pathway modulation was evaluated following WPI treatment in Pre- and Post-ExM experimental conditions in the presence of the AMPK inhibitor dorsomorphin 10 μM (compound C). In (**A**) pAMPK levels, (**B**) ERK signalling activity, (**C**) p38 MAPK signalling activity, and (**D**) mTOR levels. Cells were pretreated with dorsomorphin 10 µM prior to ExM stimulation and subsequent exposure to WPI treatment conditions. Data are expressed as mean ± SD (%) vs control from independent biological experiments performed in triplicate and normalised to the control (0%) line. * *p* < 0.05 vs. control; # *p* < 0.05 vs. ExM; bar: *p* < 0.05 vs. corresponding 8 h condition; α *p* < 0.05 vs. Pre-ExM.

After that, integrative analyses were conducted to investigate the involvement of the AMPK signalling pathway in the metabolic effects observed in C2C12-differentiated cells. Cells were pretreated with dorsomorphin (compound C; 10 µM, Merck Life Sciences, Rome, Italy), a pharmacological inhibitor of AMPK, for 30 min [34] prior to ExM stimulation and subsequent treatment with WPI under the defined experimental conditions. This experimental approach was undertaken as a preliminary and exploratory strategy to assess the contribution of AMPK signalling to the observed effects of WPI on key metabolic and stress-related endpoints, including AMPK, p38 MAPK signalling, ERK activity and modulation of mTOR pathway activation. The results of this experimental section are reported in Appendix A (Figure A1).

**Figure A2:**
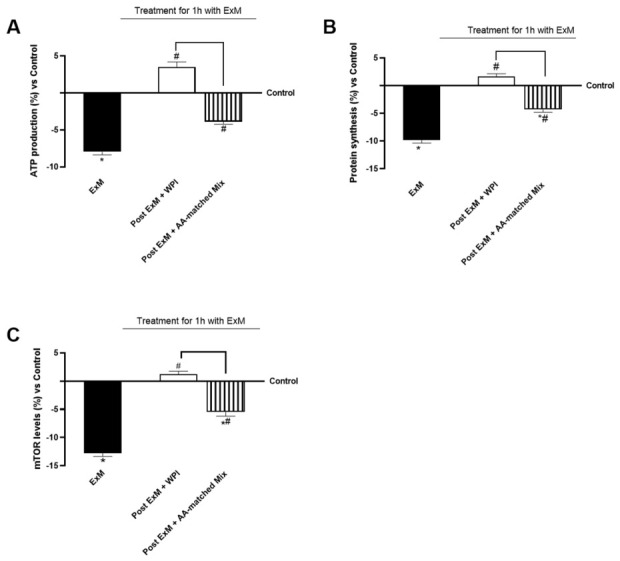
Analysis at 24 h of WPI and amino acid-matched mixture (AA-matched Mix) treatments on cellular bioenergetics and anabolic signalling pathways in C2C12-differentiated cells under Post ExM-induced metabolic stress conditions. The physiological response was evaluated following 1 h of ExM-induced stress alone, and during subsequent post-stress recovery treatments. In (**A**) ATP production levels (%); (**B**) protein synthesis rate (%); and (**C**) mTOR levels (%). Data are expressed as mean ± SD (%) vs control from five independent biological experiments performed in triplicate and normalised to the control (0%) baseline. * *p* < 0.05 vs. control; # *p* < 0.05 vs. ExM; bar *p* < 0.05 vs. AA-matched Mix.

Finally, to further investigate whether the biological effects observed following treatment with digested WPI could be primarily attributed to free amino acid availability, an amino acid-matched control mixture (AA-matched Mix) was prepared based on the principal amino acid composition of WPI. The formulation reproduced the major amino acid components reported for WPI at an equivalent final concentration of 1 mg/mL and included L-glutamic acid (17.0%), L-aspartic acid (11.0%), L-lysine (9.3%), L-leucine (9.2%), L-threonine (6.7%), L-proline (5.9%), and L-isoleucine (5.1%) with all other amino acids present at less than 5%. Amino acids were dissolved in culture medium (phenol red-free DMEM supplemented with 0% FBS, 50 IU/mL penicillin–streptomycin, and 2 mM L-glutamine), sterile filtered (MilliporeSigma, Burlington, MA, USA) through a 0.22 μm membrane, and freshly prepared prior to use. This preparation was used as an amino acid-matched control lacking intact proteins and digestion-derived peptide fractions. C2C12 cells exposed to ExM were treated either with digested WPI or with the amino acid-matched control preparation. ATP production, OPP-based protein synthesis and mTOR levels were subsequently evaluated to determine whether the observed biological effects could be explained solely by free amino acid availability. The results of this experimental section are reported in Appendix A (Figure A2).

### 2.5. Cell Viability (MTT Assay)

Cell viability was assessed using an MTT assay kit (Merck Life Science, Rome, Italy) as previously described [35]. Absorbance was measured at 570 nm with background subtraction at 690 nm. Data were normalised to untreated control cells and expressed as percentage variation. Results represent mean ± standard deviation (SD) from five independent experiments performed in triplicate.

### 2.6. ROS Production

Superoxide production was measured using a cytochrome C reduction assay [36]. Absorbance at 550 nm was recorded and results expressed as nanomoles of reduced cytochrome C per microgram of protein, normalised to control cells. Results represent mean ± standard deviation (SD) from five independent experiments performed in triplicate.

### 2.7. Annexin V Quantification

Annexin V levels were quantified using a commercially available ELISA kit validated for murine samples (Bio-Techne/Novus Biologicals, Centennial, CO, USA), according to the manufacturer’s instructions. Briefly, cell lysates or culture supernatants were collected and processed following standard procedures. Absorbance was measured at 450 nm, with correction at 570 nm. Results are expressed as mean ± SD (%) relative to control from five independent experiments performed in triplicate.

### 2.8. LDH Release Assay

Lactate dehydrogenase (LDH) release into the culture supernatant was measured as an indicator of cell membrane damage, following a specific procedure reported in the literature [37]. Briefly, cells were seeded in 96-well plates and incubated under standard conditions (37 °C, 5% CO_2_, and 95% humidity) for 24 h to allow cell attachment and stabilisation prior to treatment. After treatment, maximum LDH release was achieved by adding lysis buffer and incubating for 45 min at 37 °C. Plates were centrifuged, and aliquots of culture supernatants (50 μL) were transferred to a new plate, avoiding cell debris. An equal volume of LDH reaction mixture was added, and samples were incubated at room temperature for 10–30 min protected from light. The reaction was stopped, and absorbance was measured at 490 nm. LDH activity in the supernatant was directly proportional to membrane damage and cytotoxicity. Results are expressed as mean ± SD (%) relative to control (baseline, 0% line) from five independent experiments performed in triplicate.

### 2.9. Irisin Quantification

Irisin levels were measured using a sandwich ELISA kit (FineTest, Wuhan, China) [38]. Absorbance was measured at 450 nm, and concentrations were calculated from a standard curve. Data were normalised to untreated controls and expressed as mean ± SD from five independent experiments.

### 2.10. TNF-α Quantification

TNF-α levels were measured by ELISA (Merck Life Science, Rome, Italy) [31] and expressed as percentage variation relative to untreated controls. Data represent mean ± SD from five independent experiments.

### 2.11. IL-6 Quantification

IL-6 levels were quantified using ELISA (FineTest, Wuhan, China) [39], normalised to control values, and expressed as mean ± SD.

### 2.12. IL-17 Quantification

IL-17 levels were measured using ELISA (FineTest, Wuhan, China) [40] and expressed as mean ± SD relative to untreated controls.

### 2.13. Lactate Production

Intracellular lactate was quantified using a colourimetric assay (BioVision, Milpitas, CA, USA) [41], normalised to total protein content, and expressed as mean ± SD.

### 2.14. Phospho-p38 MAPK

Phosphorylated p38 MAPK levels were measured using ELISA (Abcam, Cambridge, UK) [42] and expressed as mean ± SD (%) relative to untreated controls.

### 2.15. ERK Activity

ERK activation was assessed using ELISA (Thermo Fisher Scientific, Milan, Italy) [35] and expressed as mean ± SD (%) relative to untreated controls.

### 2.16. ATP Measurement

ATP levels were determined using a luminescent assay (Calbiochem, San Diego, CA, USA) [31], normalised to total protein content, and expressed as mean ± SD (%) relative to untreated controls.

### 2.17. AMPK Determination

AMPK phosphorylation levels in C2C12-differentiated cell lysates were measured using ELISA (Thermo Fisher, Milan, Italy) [43] and expressed as mean ± SD (%) relative to untreated controls.

### 2.18. Glycogen Measurement

Glycogen levels were quantified using a colourimetric assay (BioVision, Life Research, Scoresby, Victoria, Australia) and normalised to total protein content. Results are expressed as mean ± SD relative to untreated controls.

### 2.19. mTOR Determination

mTOR levels were measured using a sandwich ELISA (FineTest, Wuhan, China) and normalised to total protein content. Results are expressed as mean ± SD (%) relative to untreated controls.

### 2.20. Protein Synthesis (OPP Assay)

Protein synthesis was assessed using O-propargyl-puromycin incorporation (assay kit; Abcam, Cambridge, UK) [44]. Fluorescence intensity was measured and normalised to total protein content. Results are expressed as mean ± SD (%) relative to untreated controls.

### 2.21. Calcium and Magnesium Fluxes

Intracellular Ca^2+^ and Mg^2+^ dynamics were measured using Fura-2 AM (Merck Life Science, Milan, Italy) and Mag-fura-2 AM fluorescent probes (Furaptra, Biotium, Fremont, CA, USA). The physiological threshold was defined based on control baseline values obtained under untreated conditions. Fluorescence ratios were calculated and expressed relative to untreated controls.

### 2.22. Western Blot

C2C12-differentiated cells were lysed on ice using Complete Tablet Buffer (Roche, Basel, Switzerland) supplemented with 1 mM PMSF, 2 mM sodium orthovanadate (Na_3_VO_4_), a 1:50 dilution of phosphatase inhibitor cocktail, and a 1:200 dilution of protease inhibitor cocktail, following established protocols. Protein extracts (35 µg) were separated on 8% and 10% sodium dodecyl sulfate-polyacrylamide gel electrophoresis (SDS-PAGE) gels and transferred onto polyvinylidene fluoride (PVDF) membranes (GE Healthcare Europe GmbH, Milan, Italy). Membranes were incubated overnight at 4 °C with primary antibodies (all 1:500; Santa Cruz Biotechnology, Santa Cruz, CA, USA) against pAMPK (pAMPK Thr172, rabbit polyclonal Cat. No. sc-3524), AMPK (mouse monoclonal Cat. No. sc-74461), p-mTOR (p-mTOR Ser2448, mouse monoclonal Cat. No. sc-293133), mTOR (mouse monoclonal Cat. No. sc-517464). Secondary antibodies used were HRP-conjugated anti-mouse IgG (1:5000; Cat. No. A9044, Merck Life Science, Rome, Italy) and HRP-conjugated anti-rabbit IgG (1:5000; Cat. No. A0545, Merck Life Science, Rome, Italy). Protein loading was verified using an anti-β-actin mouse monoclonal antibody (1:4000; Cat. No. A5441, Merck Life Science, Rome, Italy), which was used as a loading control for normalisation of total protein expression. Phosphorylated protein levels were normalised to their corresponding total protein levels, and then further normalised to β-actin where appropriate. Image Lab^TM^ Software (version 5.2.1; Bio-Rad, Hercules, CA, USA) analysed the densitometry and band intensities. Representative blot images were selected from experiments showing results consistent with the overall quantitative analysis obtained from independent biological replicates. Results are expressed as mean ± SD (%) relative to untreated controls.

### 2.23. Fourier Transform Infrared (FT-IR) Spectroscopy Analysis

FT-IR spectroscopy was performed on the native WPI sample and on WPI after incubation with the C2C12-differentiated cell line. Precisely, digested WPI samples were obtained through a two-step process: an initial in vitro enzymatic digestion to mimic oral intake using the INFOGEST protocol (method reported in Appendix A), followed by incubation with C2C12-differentiated cells for 24 h. Then, both native WPI powder and WPI samples recovered post-cellular exposure (referred to as WPI after incubation with C2C12 cells) were analysed without further processing. Spectra were acquired using an FT-IR spectrometer, Bruker Alpha II instrument (Bruker Optics, Rosenheim, Germany), equipped with an attenuated total reflectance (ATR) accessory. Spectra were recorded in the mid-infrared range of 4000 to 400 cm^−1^ with a resolution of 4 cm^−1^, accumulating 32 scans per sample to improve the signal-to-noise ratio. The ATR crystal was cleaned between samples with ethanol and dried to avoid cross-contamination. The spectral analysis focused primarily on the Amide I (~1650 cm^−1^) and Amide II (~1540 cm^−1^) bands, characteristic of protein secondary structure, as well as on the broad stretching region corresponding to N–H and O–H bonds (3000–3700 cm^−1^). Spectral differences were evaluated qualitatively and by comparison of peak positions and intensities.

### 2.24. Statistical Analysis

Data are expressed as mean ± SD from at least five independent biological experiments (*n* = 5). For each biological replicate, measurements were performed in technical triplicate and averaged prior to statistical analysis; thus, each data point used for statistical testing corresponds to one biological replicate.

Western blot data are expressed as mean ± SD from at least three independent biological experiments (*n* = 3), each performed in technical triplicate. Band intensities were quantified by densitometric analysis and averaged per biological replicate prior to statistical evaluation. Phosphorylated proteins were normalised to their corresponding total protein levels, while total protein expression was normalised to β-actin as a loading control. All experimental data, including colourimetric/functional assays and Western blot analyses, were normalised to the untreated control. The untreated control was used as the reference condition and defined as 0% variation, according to the following formula:

$$\%\mathrm{Variation}=\left(\frac{{\mathrm{Normalized}\;\mathrm{OD}}_{\mathrm{treated}}}{{\mathrm{Normalized}\;\mathrm{OD}}_{\mathrm{control}}}-1\right)\times 100$$

Therefore, untreated control values were set as the reference condition (0%), with positive and negative values representing increases or decreases relative to baseline, respectively.

Fold change values for the remaining experimental endpoints were calculated as the ratio between treated and reference values (Treated/Reference). Percentage variation relative to the reference condition was then derived using the following formula: ((Treated − Reference)/Reference) × 100.

Statistical analysis was performed using GraphPad Prism (version 10.2.3; GraphPad Software, Inc., San Diego, CA, USA). Normality of data distribution was assessed using the Shapiro–Wilk test. Differences among groups were evaluated using one-way analysis of variance (ANOVA) followed by Tukey’s post hoc multiple comparison test. A *p*-value < 0.05 was considered statistically significant.

## 3. Results

### 3.1. Dose–Response Study of WPI on C2C12-Differentiated Cells

**Figure 2:**
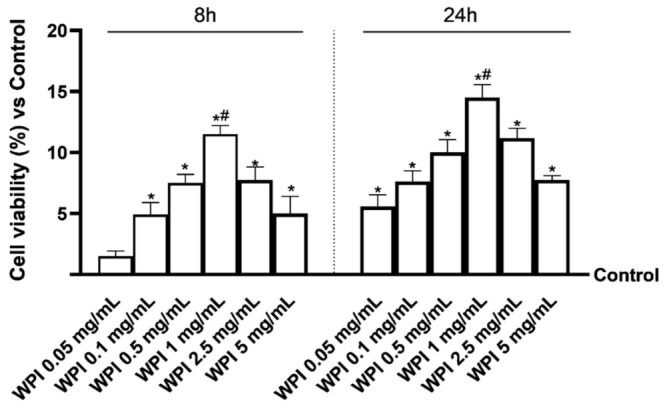
Dose–response effect of WPI on C2C12-differentiated cell viability. C2C12 cells were treated with increasing concentrations of WPI (0.05–5 mg/mL) for 8 and 24 h. Cell viability was assessed by MTT assay and normalised to untreated control cells (0% reference). Data are presented as mean ± SD of five independent experiments, each performed in technical triplicate. * *p* < 0.05 vs. control; # *p* < 0.05 vs. other concentrations.

The initial phase of the study assessed the dose–response effects of WPI on C2C12-differentiated cell viability after 8 and 24 h of treatment (0.05–5 mg/mL). As shown in Figure 2, MTT assay results, normalised to untreated control cells (0% reference), demonstrated a dose- and time-dependent response up to 1 mg/mL, followed by a decrease at higher concentrations. WPI alone did not significantly alter baseline cellular parameters across the tested conditions, supporting its use as a modulator primarily under stress.

At 8 h, cell viability gradually increased across the tested concentrations, peaking at 1 mg/mL (+11.5% compared to control; *p* < 0.05). At higher concentrations (2.5 and 5 mg/mL), viability levels decreased relative to the peak but remained significantly higher than those of the untreated control (*p* < 0.05).

A similar trend was seen at 24 h, with a more noticeable effect. The highest value was again observed at 1 mg/mL (+14.5% compared to control; *p* < 0.05), followed by a decrease at higher concentrations.

Overall, these data indicate that WPI is well tolerated across the tested concentration range and support choosing 1 mg/mL as the optimal concentration for subsequent experiments.

### 3.2. Pre- and Post-Exposure Effects of WPI on C2C12-Differentiated Cells Under ExM Stress Condition

**Figure 3:**
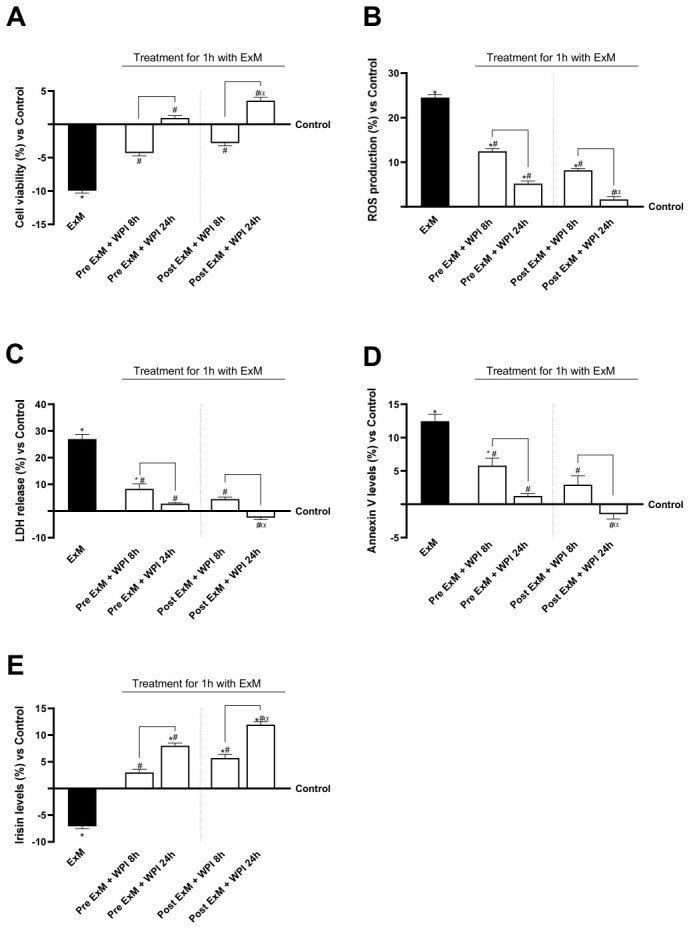
Effect of WPI (1 mg/mL) on cell viability, ROS production, and irisin levels following ExM-induced stress. C2C12-differentiated cells were exposed to 1 h of ExM and treated with WPI under Pre-ExM or Post-ExM conditions for 8 and 24 h. In (**A**), percentage change in cell viability; in (**B**), ROS production; in (**C**), LDH release determination; in (**D**), Annexin V levels; in (**E**), analysis of irisin protein levels. Data are expressed as mean ± SD of five independent experiments, each performed in technical triplicate, and normalised to untreated control cells (0% reference). * *p* < 0.05 vs. control; # *p* < 0.05 vs. ExM; bar *p* < 0.05 vs. corresponding 8 h condition; α *p* < 0.05 vs. Pre-ExM.

Following dose–response assessment, the effects of WPI (1 mg/mL) were examined in an in vitro model of ExM-induced metabolic stress (Figure 3). C2C12-differentiated cells were exposed to ExM for 1 h and treated according to two protocols: Pre-ExM and Post-ExM, with incubation periods of 8 or 24 h.

As shown in Figure 3A, ExM exposure significantly decreased cell viability (approximately −10% compared to untreated control; *p* < 0.05). Both treatment protocols partly mitigated this reduction. The Pre-ExM condition resulted in a moderate recovery in cell viability, particularly at 24 h. A greater effect was observed in the Post-ExM condition, particularly at 24 h, where viability values approached or slightly exceeded control levels, though not always statistically significant compared to untreated cells.

Analysis of oxidative stress (Figure 3B) showed that ExM exposure significantly increased ROS production (+24.5% compared to control; *p* < 0.05). WPI treatment was associated with lower ROS levels under both protocols (*p* < 0.05 vs. ExM), with a time-dependent pattern. The reduction was more pronounced at 24 h, with ROS levels approaching baseline, especially in the Post-ExM condition.

Additional data were obtained to evaluate the cytotoxic potential of WPI (1 mg/mL). Figure 3C shows LDH release into the culture medium following the two treatment protocols. Exposure to ExM alone for 1 h induced a marked pro-oxidant response, with a 24.5% increase in ROS levels compared to the untreated control (*p* < 0.05). Treatment with WPI (1 mg/mL), administered according to both protocols, was associated with significantly reduced ExM-induced LDH release (*p* < 0.05 vs. ExM), with a time-dependent pattern (*p* < 0.05 vs. ExM). This reduction was more pronounced after 24 h than after 8 h (*p* < 0.05). Notably, in the 24 h Post ExM + WPI condition, LDH levels are lower than baseline values and slightly lower than those observed in the untreated control. Overall, the 24 h Post ExM + WPI treatment showed a 1.10-fold decrease compared to ExM, as well as reductions of 72%, 18%, and 49% compared to the 8 h Pre ExM + WPI, 24 h Pre ExM + WPI, and 8 h Post ExM + WPI conditions, respectively (*p* < 0.05).

Similar data were also obtained for intracellular Annexin V levels (Figure 3D). ExM alone for 1 h induced a significant 12.5% increase in Annexin V levels compared to the untreated control (*p* < 0.05). Treatment with WPI (1 mg/mL), according to both protocols, was associated with reduced ExM-induced Annexin V levels, in a time-dependent trend (*p* < 0.05 vs. ExM). Also in this parameter, the reduction was more pronounced after 24 h than after 8 h (*p* < 0.05). The Post ExM + WPI at 24 h condition, Annexin V levels were close to baseline values. Overall, Post ExM + WPI at 24 h treatment showed a 1.12-fold decrease compared to ExM, as well as reductions of 74%, 15%, and 55% compared to the 8 h Pre ExM + WPI, 24 h Pre ExM + WPI, and 8 h Post ExM + WPI conditions, respectively.

Irisin levels (Figure 3E) were significantly decreased after ExM exposure (around −7% compared to control; *p* < 0.05). WPI treatment countered this effect, aiding the recovery of irisin levels in both experimental conditions. This response followed a time-dependent pattern, with higher levels observed at 24 h compared to 8 h (*p* < 0.05). The Post-ExM condition at 24 h showed the most consistent recovery of irisin levels among the tested conditions.

Overall, these results suggest that WPI influences cellular responses to ExM-induced stress, with a significant modulation under the Post-ExM protocol, especially at 24 h.

### 3.3. Modulation of Inflammatory Cytokines Under ExM-Induced Stress

**Figure 4:**
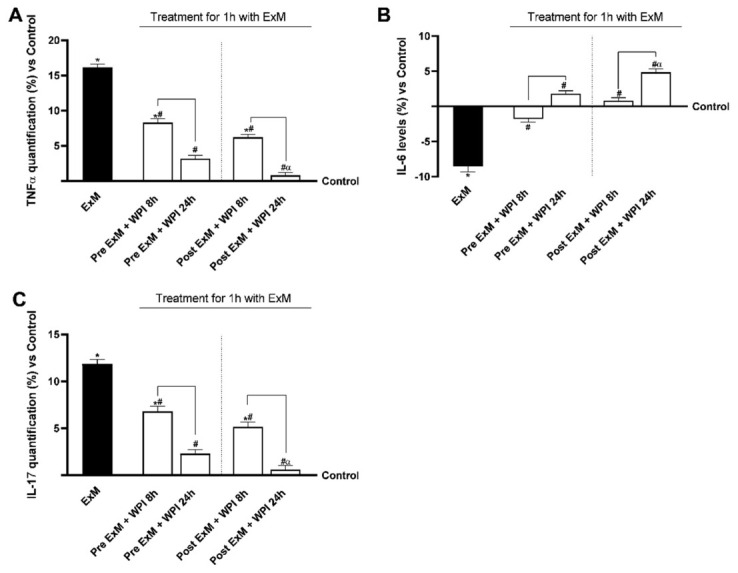
Effect of WPI (1 mg/mL) on cytokine levels following ExM-induced stress. C2C12-differentiated cells were exposed to 1 h of ExM and treated with WPI under Pre-ExM or Post-ExM conditions for 8 and 24 h. (**A**) TNF-α levels; (**B**) IL-6 levels; (**C**) IL-17 levels. Data are expressed as mean ± SD of five independent experiments, each performed in technical triplicate, and normalised to untreated control cells (0% reference). * *p* < 0.05 vs. control; # *p* < 0.05 vs. ExM; bar: *p* < 0.05 vs. corresponding 8 h condition; α *p* < 0.05 vs. Pre-ExM.

In this experimental phase, changes in key cytokines related to inflammatory and metabolic responses were assessed (Figure 4). Exposure of C2C12-differentiated cells to ExM for 1 h led to a notable increase in TNF-α (+16%) and IL-17 (+12%) levels, along with a decrease in IL-6 (−8%) compared to untreated control cells (*p* < 0.05). WPI treatment (1 mg/mL) modulated these responses in both Pre-ExM and Post-ExM conditions. TNF-α and IL-17 levels (Figure 4A,C) were lower following WPI treatment compared with ExM condition (*p* < 0.05 vs. ExM), displaying a time-dependent pattern. This effect was more evident at 24 h, especially in the Post-ExM condition, where cytokine levels approached those of untreated controls.

Regarding IL-6 (Figure 4B), WPI treatment was associated with higher IL-6 levels compared with ExM conditions in both experimental protocols. This effect was more pronounced at 24 h, with the Post-ExM condition showing the highest values among the tested conditions (*p* < 0.05 vs. ExM).

Overall, these findings suggest that WPI influences cytokine responses under ExM-induced stress, with a more consistent effect seen in the Post-ExM condition at 24 h.

### 3.4. Modulation of Signalling Pathways and Energy Metabolism by WPI Under ExM-Induced Stress

**Figure 5:**
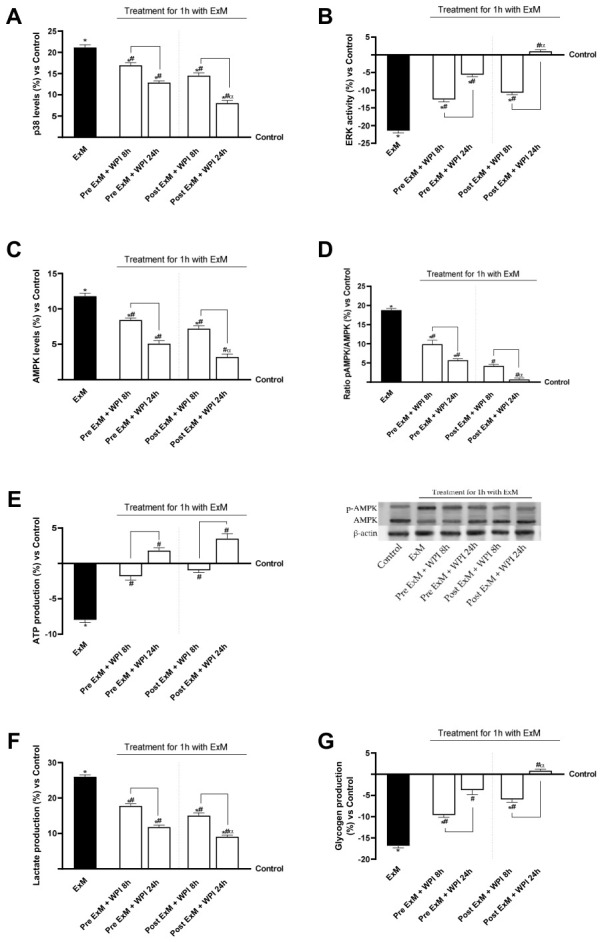
Effect of WPI (1 mg/mL) on signalling pathways and energy metabolism following ExM-induced stress. C2C12-differentiated cells were exposed to 1 h of ExM and treated with WPI under Pre-ExM or Post-ExM conditions for 8 and 24 h. In (**A**) percentage change in p38 levels; in (**B**) ERK activity; in (**C**) AMPK levels; in (**D**) densitometric analysis of p-AMPK/AMPK ratio with a representative image below after Western blot; in (**E**) ATP production; in (**F**) lactate production; in (**G**) Glycogen production. Data are expressed as mean ± SD of five independent experiments, each performed in technical triplicate, and normalised to untreated control cells (0% reference). Western blot data are presented as mean ± SD (%) from three independent experiments, each performed in triplicate * *p* < 0.05 vs. control; # *p* < 0.05 vs. ExM; bar *p* < 0.05 vs. corresponding 8 h condition; α *p* < 0.05 vs. Pre-ExM.

To further explore the mechanisms behind the observed cellular responses, key signalling pathways and metabolic parameters were assessed (Figure 5). Exposure to ExM caused a significant increase in p38/MAPK and AMPK phosphorylation (+21% and +12%, respectively; *p* < 0.05 vs. control), while ERK activity was significantly lower compared to untreated cells.

At the metabolic level, ExM lowers ATP production (−8%) and glycogen content (−17%) and higher lactate levels (+26%) (*p* < 0.05 vs control), indicating an altered bioenergetic state.

WPI treatment (1 mg/mL) modulated these changes under both Pre-ExM and Post-ExM conditions. Specifically, WPI treatment was associated with reduced p38 and AMPK activation and with modulation of ERK activity compared with ExM-treated cells (*p* < 0.05). Simultaneously, WPI treatment was associated with improvements in metabolic parameters, including higher ATP and glycogen levels and lower lactate accumulation (*p* < 0.05 vs. ExM).

These effects demonstrated a time-dependent pattern, with differences more evident at 24 h. The Post-ExM condition at 24 h showed changes in both signalling and metabolic parameters, with values nearing those seen in untreated control cells.

Overall, these findings suggest that WPI modulates signalling pathways and bioenergetic responses under ExM-induced stress conditions.

Additional data regarding AMPK, ERK, and p38 signalling pathways in the presence of the AMPK pharmacological inhibitor, dorsomorphin, are reported in Appendix A (Figure A1), supporting the exploratory analysis of pathway modulation under AMPK inhibition. AMPK phosphorylation was partially reduced following inhibitor treatment, while ERK and p38 responses remained detectable and preserved a similar time-dependent pattern in Pre- and Post-ExM WPI treatments.

Comparative data, at 24 h, between digested WPI and an AA-matched Mix control are also reported in Appendix A (Figure A2). As shown, Post-ExM + WPI resulted in higher ATP production and protein synthesis than the AA-matched mix (*p* < 0.05). The AA-matched Mix control induced a partial recovery of cellular bioenergetic and anabolic parameters, whereas digested WPI was associated with a more pronounced restoration toward baseline levels, suggesting that factors beyond free amino acid availability may contribute to the observed responses under the experimental conditions.

### 3.5. Modulation of mTOR Signalling and Protein Synthesis

**Figure 6:**
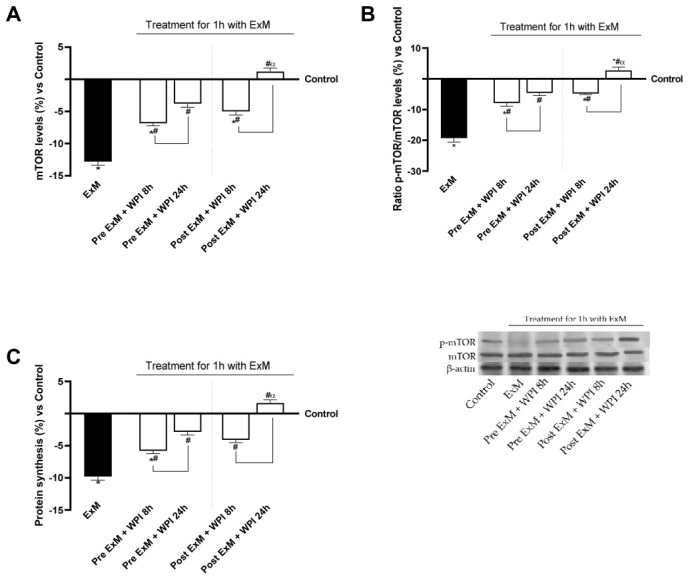
Effect of WPI (1 mg/mL) on mTOR signalling and protein synthesis following ExM-induced stress. C2C12-differentiated cells were exposed to 1 h of ExM and treated with WPI under Pre-ExM or Post-ExM conditions for 8 and 24 h. In (**A**), percentage change in mTOR protein levels; in (**B**), densitometric analysis of p-mTOR/mTOR ratio with an example image below after Western blot; in (**C**), analysis of protein synthesis rate. Data are expressed as mean ± SD of five independent experiments, each performed in technical triplicate, and normalised to untreated control cells (0% reference). Western blot data are presented as mean ± SD (%) from three independent experiments, each performed in triplicate. * *p* < 0.05 vs. control; # *p* < 0.05 vs. ExM; bar: *p* < 0.05 vs. corresponding 8 h condition; α *p* < 0.05 vs. Pre-ExM.

The activation of the mTOR pathway and the rate of de novo protein synthesis were evaluated under different experimental conditions (Figure 6). Exposure of C2C12-differentiated cells to stress induced by ExM caused a notable decrease in both mTOR activation (−13%) and protein synthesis (−10%) compared to untreated control cells (*p* < 0.05). WPI treatment (1 mg/mL) influenced these changes in a time-dependent manner across both Pre-ExM and Post-ExM protocols. While the Pre-ExM condition showed partial recovery of mTOR signalling and protein synthesis, the Post-ExM condition exhibited a more significant effect. After 24 h, the Post-ExM treatment showed the most consistent recovery of both mTOR activation and protein synthesis compared to ExM-treated cells (*p* < 0.05). In this context, values were nearly matched to or slightly exceeded those of untreated controls, though not always statistically significant. Regarding Western blot analysis of p-mTOR/mTOR expression, Post ExM + WPI treatment at 24 h led to a marked increase in mTOR activation compared to ExM-treated cells (*p* < 0.05).

Overall, these findings suggest that WPI influences anabolic signalling and protein synthesis under ExM-induced stress, with a more notable effect in the Post ExM condition at 24 h.

Additional data regarding mTOR signalling under pharmacological AMPK inhibition (dorsomorphin) are reported in Appendix A (Figure A2), supporting the exploratory analysis of pathway modulation. mTOR levels show a time-dependent modulation under ExM conditions, with a partial shift toward baseline levels following WPI treatment.

Comparative data, at 24 h, between digested WPI and an AA-matched Mix control are also reported in Appendix A on mTOR levels (Figure A2). As shown, Post-ExM + WPI resulted in higher mTOR levels compared with the amino AA-matched Mix (*p* < 0.05).

### 3.6. Modulation of Ca^2+^/Mg^2+^ Fluxes Under ExM-Induced Stress

**Figure 7:**
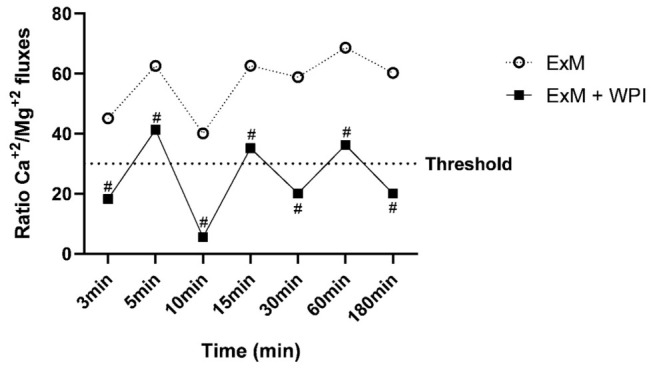
Effect of WPI (1 mg/mL) on Ca^2+^/Mg^2+^ flux ratio following ExM-induced stress. C2C12-differentiated cells were monitored for 180 min after ExM exposure, with or without WPI treatment. The dotted line represents the physiological threshold. Data are expressed as mean ± SD of five independent experiments, each performed in triplicate. # *p* < 0.05 vs. ExM.

Integrative analyses of the Ca^2+^/Mg^2+^ flux ratio were conducted over a 3–180 min period following ExM exposure (Figure 7). ExM treatment caused an immediate increase in Ca^2+^/Mg^2+^ ratio, which remained above throughout the defined physiological threshold throughout the observation period, with peaks observed at 5, 15, and 60 min. WPI treatment (1 mg/mL) was associated with modulation of these ionic dynamics. In WPI-treated cells, the Ca^2+^/Mg^2+^ ratio was significantly lower compared to ExM-treated cells at all time points (*p* < 0.05 vs. ExM). Additionally, the ratio mostly stayed below the physiological threshold and stabilised over time, especially at later time points (up to 180 min). Overall, these findings suggest that WPI affects Ca^2+^/Mg^2+^ fluxes under ExM-induced stress conditions.

### 3.7. FT-IR Analysis of WPI Structural Changes After C2C12 Incubation

**Figure 8:**
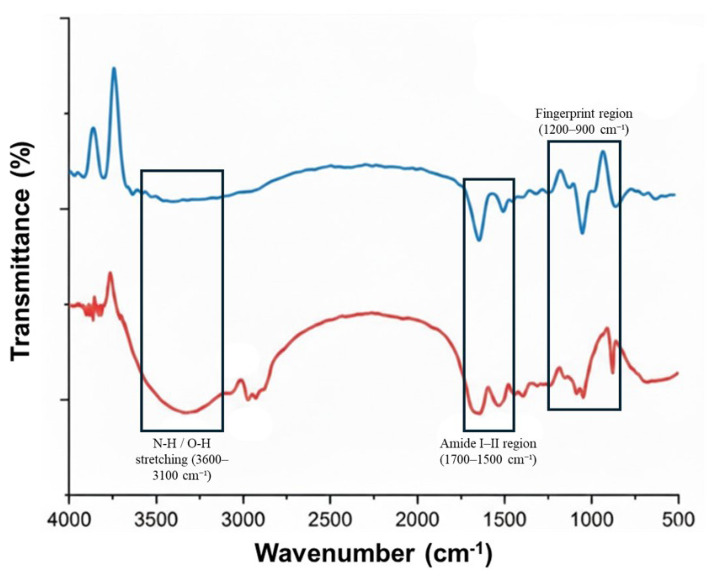
Comparative FT-IR spectra of native WPI (red) and digested WPI after incubation with C2C12-differentiated cells (blue). Spectra highlight changes in key regions: N–H/O–H stretching (3600–3100 cm^−1^) shows increased intensity, while Amide I–II bands (1700–1500 cm^−1^) exhibit shifts and broadening, indicating structural remodelling of WPI after cell incubation. Data are representative of three independent experiments.

FT-IR spectra (Figure 8) were acquired for native WPI (red trace) and WPI digested after 24-h incubation with C2C12-differentiated cells (blue trace) to investigate potential structural modifications induced by the cellular environment. Native WPI exhibited well-defined Amide I and Amide II bands, with maxima at approximately 1650 cm^−1^ and 1540 cm^−1^, respectively. These bands are consistent with the presence of ordered secondary structures, primarily α-helices and β-sheets, indicating that the protein matrix maintained high conformational integrity. The N–H/O–H stretching region (3000–3700 cm^−1^) showed a broad but relatively low-intensity signal, reflecting limited hydrogen bonding activity in the native protein. Minor features in the 1400–1450 cm^−1^ region, corresponding to C–H bending vibrations, were also observed, consistent with side-chain conformations of the intact protein.

Following incubation with C2C12-differentiated cells, the FT-IR spectrum of digested WPI exhibited several notable, reproducible modifications. The Amide I band shifted from 1650 cm^−1^ to approximately 1645 cm^−1^ and broadened, suggesting increased heterogeneity in the secondary structure. Similarly, the Amide II band shifted from 1540 cm^−1^ to around 1535 cm^−1^ and displayed a modest increase in intensity. These changes are consistent with structural rearrangements in the protein backbone. Additionally, the N–H/O–H stretching region displayed a marked increase in intensity, particularly between 3200 and 3500 cm^−1^, consistent with changes in hydrogen bonding interactions and possible increased exposure of polar groups within the protein structure. Subtle changes were also observed in the C–H bending region, suggesting minor adjustments in side-chain conformations. These observations should be considered exploratory, but provide supportive evidence of structural adaptation of WPI in the cellular environment.

## 4. Discussion

Understanding the molecular mechanisms behind post-exercise recovery is crucial for clarifying cellular adaptation to metabolic stress in skeletal muscle [45,46,47]. The preliminary dose–response characterization suggested that the concentration of 1 mg/mL represents an intermediate dose commonly reported in the literature for C2C12 cells and appears compatible with maintaining cell viability under comparable experimental conditions. Within this context, this concentration may represent a reasonable balance between biological relevance and experimental feasibility in skeletal muscle cell models [24,25].

After that, in this study, we assessed the effects of WPI (1 mg/mL) in a pharmacological ExM in vitro system designed to reproduce selected aspects of metabolic stress and found that it modulates multiple pathways involved in the cellular stress response, including inflammatory, metabolic, and ionic processes. This model allows the controlled induction of metabolic stress-related signalling pathways without the confounding influence of mechanical contraction [23,26,48].

Our findings indicate that ExM exposure was associated with a cellular stress response characterised by increased oxidative and inflammatory markers [49,50]. The use of a 1 h stimulation window is consistent with commonly employed in vitro models of acute chemical exercise-mimetic stress, which are generally intended to capture early signaling events prior to the onset of transcriptional and metabolic remodeling [23,48,51]. Although mechanical stretch models are commonly used to study skeletal muscle mechanotransduction [52], they primarily reproduce the biomechanical component of exercise and only partially reflect the integrated metabolic and biochemical responses associated with exercise-induced stress. Key events such as AMPK activation, ROS production, intracellular calcium fluctuations, and metabolic stress responses are not fully captured by stretch-based systems [23]. For this reason, the ExM model was selected as it better reproduces the metabolic and bioenergetic alterations relevant to the aims of this study.

Elevated ROS levels and activation of p38 MAPK are well-known indicators of cellular stress and damage [53,54]. Under these conditions, WPI treatment was associated with reduced ROS production and pro-inflammatory cytokines (TNF-α and IL-17), as well as preservation of cell viability. WPI and its derived bioactive peptides have been shown to enhance cellular antioxidant defences, reduce oxidative damage, and modulate apoptotic pathways under stress conditions [24]. The observed reduction in LDH release and Annexin V levels was associated with changes in membrane integrity and apoptosis-related markers following ExM-induced stress [24]. In line with previous reports in C2C12, protein supplementation has been associated with reduced levels of oxidative stress and apoptosis markers, supporting a role for WPI in maintaining muscle cell homeostasis under damaging conditions [55]. The more pronounced effects observed after 24 h further suggest that sustained exposure to WPI may be associated with enhanced activation of cellular protective responses.

Attention was paid to irisin, a myokine involved in metabolic regulation and cellular adaptation [56]. In our model, ExM exposure decreased irisin levels, whereas WPI treatment was associated with a recovery of its expression. However, given the ongoing debate surrounding the quantification and physiological relevance of irisin in in vitro systems, these findings should be interpreted with caution. The modulation of IL-6 observed in this study may reflect complex, context-dependent roles, as IL-6 can exert both pro- and anti-inflammatory effects depending on the cellular environment [57,58]. Within the present in vitro model, IL-6 should be interpreted as a component of the broader stress-related signalling network rather than as an indicator of a specific adaptive or myokine-like response.

Previous studies have reported that whey protein intake may influence cytokine profiles in both in vitro and clinical settings [59,60], suggesting a potential role in regulating inflammatory pathways. Although IL-6 has been described as a myokine in the context of skeletal muscle contraction [61], such physiological interpretations are not directly applicable to the present pharmacologically induced in vitro model. Our results are consistent with previous observations [59,60]; however, the observed changes in IL-6 levels should be interpreted considering the limitations of the experimental system and considered as part of a broader response to inflammatory and metabolic stress. This interpretation remains associative and does not support conclusions regarding direct regulatory effects on downstream signalling pathways in this in vitro model.

The comparison between Pre-ExM and Post-ExM protocols provides insight into temporal aspects of cellular responses. Pre-treatment with WPI, under 1 h-ExM conditions, may be associated with partial protection against stress-induced damage, possibly by providing amino acids and its antioxidant capacity [62,63]. However, a more pronounced effect was observed under the Post-ExM condition within this experimental context, suggesting that WPI was associated with modulation of cellular homeostasis-related responses following metabolic stress within this in vitro context. This is supported by previous findings showing that whey-derived peptides can reduce oxidative and inflammatory responses in stressed C2C12 cells [62].

The modulation of energy metabolism is a key aspect of these responses. The ExM mixture, containing AICAR and 8-Bromo-cAMP, modulates a catabolic state characterised by AMPK activation and decreased ATP levels [64,65]. Consistent with previous reports, AMPK activation acts as a regulatory mechanism that shifts cellular metabolism towards energy conservation [66], leading to lower ATP and glycogen levels and increased lactate accumulation [67]. In this context, WPI treatment was associated with modulation of AMPK activation, partial restoration of ATP and glycogen levels, and decreased lactate accumulation. These findings align with studies suggesting that amino acids may influence mitochondrial metabolism and intracellular energy-related pathways [25,67,68].

Meanwhile, the restoration of ERK activity seems to play a significant role in the observed responses. ERK signalling influences cell survival, metabolism, and stress adaptation [69,70]. In our model, WPI treatment was associated with changes in ERK activity, which, together with modulation of cellular energetic status, may contribute to the regulation of mTOR-related anabolic signalling pathways [71]. Notably, the Post-ExM condition at 24 h exhibited the most consistent effects on these parameters, which may indicate that the temporal sequence of nutrient exposure relative to the stress stimulus differentially modulated anabolic signalling pathways within the present experimental model. Importantly, the integration of a standardised in vitro digestion protocol (INFOGEST) suggests that the observed effects may be attributable not only to intact WPI but also, and more plausibly, to digestion-derived peptide fractions and free amino acids, which are considered the bioaccessible components potentially responsible for the biological activity observed in the present model.

The idea of a “window of plasticity” can help explain these results [72]. Although a time-dependent attenuation of ExM-induced stress cannot be completely excluded, the differential responses observed at 24 h suggest that the effects detected in the Post-ExM condition were not exclusively attributable to passive decay of the stress stimulus [48]. Rather, the findings indicate that the temporal relationship between ExM exposure and WPI administration differentially modulates cellular metabolic and anabolic signalling pathways within this in vitro system [73,74]. These observations may reflect altered cellular responsiveness to nutrient-derived signals following pharmacologically induced metabolic stress, although no direct conclusions regarding physiological post-exercise recovery or nutrient timing strategies can be inferred from the present model [25,75,76]. In this context, the amino acid profile of WPI, particularly its leucine content, may contribute to the activation of mTOR signalling and protein synthesis [77,78]. Nevertheless, it is important to recognise that the absence of a comparator group (such as isolated amino acids like leucine or BCAAs) prevents distinguishing between the general effects of amino acids and the specific properties of WPI.

Pharmacological inhibition of AMPK with dorsomorphin (Compound C), as reported in Appendix A, showed a partial reduction in AMPK phosphorylation, while ERK and p38 signalling responses remained detectable and preserved their overall time-dependent pattern under Pre- and Post-ExM conditions. A similar time-dependent modulation was observed for mTOR signalling, with a trend toward baseline levels following WPI treatment. Overall, these exploratory observations suggest that AMPK signalling may contribute to the regulation of the observed metabolic, anabolic, and stress-related responses, without fully accounting for downstream pathway modulation.

Ionic homeostasis adds an extra layer of regulation. ExM exposure, especially through ionomycin, disrupted the Ca^2+^/Mg^2+^ balance, which is vital for numerous cellular processes [28]. It should be noted that ionomycin was associated with a non-physiological elevation of intracellular Ca^2+^ levels that does not fully replicate the tightly regulated calcium transients associated with excitation–contraction coupling in skeletal muscle [79]. Therefore, ionomycin should be considered a pharmacological tool to induce calcium-dependent signalling rather than a physiological mimic of muscle contraction. In contrast to calcium-dependent stress signalling, magnesium plays a key role as a cofactor for ATP-dependent enzymes and in preserving ribosomal stability during protein synthesis [57,80,81]. In this study, WPI treatment was associated with modulation of Ca^2+^/Mg^2+^ fluxes, suggesting a possible role in maintaining ionic balance under stress conditions [75].

The comparison between Pre-ExM and Post-ExM WPI treatments highlights temporal differences in cellular responsiveness to WPI exposure relative to the exercise-mimetic stimulus, suggesting that the temporal context of exposure may differentially influence inflammatory, metabolic, and anabolic signalling pathways within this in vitro model. Given the pharmacologically induced nature of the exercise-mimetic stress model, the Pre-ExM/Post-ExM design should be interpreted as an experimental approach to investigate timing-dependent cellular responses within a controlled in vitro environment, rather than as a direct representation of physiological nutrient timing or post-exercise recovery processes in vivo.

FT-IR analysis provides further structural information following incubation under the experimental conditions. The observed shifts and broadening of the Amide I and II bands after incubation suggest that WPI undergoes structural rearrangements within the cellular environment, possibly reflecting partial unfolding or enzymatic processing of the protein [82]. Such conformational modifications may reflect changes in protein secondary structure, potentially affecting its physicochemical properties and favouring the generation of peptide fragments during subsequent enzymatic processing [83]. The increased N–H/O–H stretching indicated changes in hydrogen bonding and greater exposure of polar residues, which may affect interactions with the cellular environment and subsequent processing of WPI-derived amino acids and peptides [84]. These molecular changes indicate potential structural modifications of WPI under the experimental conditions, suggesting partial structural rearrangements in the presence of a cellular environment [62]. Such structural changes may reflect time-dependent modifications of WPI under the experimental conditions observed at 24 h. These findings should be interpreted as exploratory structural observations and not as direct evidence of functional intracellular mechanisms.

Despite these results, the study has some limitations. The use of C2C12-differentiated cells and a pharmacological stress model does not fully reproduce the complexity of in vivo muscle. The absence of multicellular interactions, vascularisation, and neuromuscular input limits the ability of the model to fully recapitulate exercise-induced adaptations occurring in physiological conditions. Furthermore, although preliminary pharmacological inhibition experiments using dorsomorphin were performed to support pathway involvement, comprehensive pathway-specific inhibition analyses were not conducted, which limits the ability to fully establish mechanistic causality. The use of C2C12-differentiated cells, while advantageous for the assessment of rapid and sensitive intracellular signalling responses, represents a simplified system characterised by high metabolic plasticity and low basal activation of differentiation-related pathways. Consequently, the present findings should be interpreted primarily as early cellular signalling responses rather than as a direct representation of mature skeletal muscle physiology or long-term stress-induced cellular adaptation. The chemical ExM protocol represents a reductionist in vitro model that reproduces selected aspects of exercise-induced metabolic stress but does not fully recapitulate mechanical contraction, force generation, or the integrated systemic responses observed in vivo. It should not be interpreted as a direct surrogate of physiological exercise.

The use of a complex digested protein mixture precludes the identification of specific bioactive peptides or amino acids responsible for the observed effects, limiting mechanistic resolution. Accordingly, due to the absence of a comparator protein or amino acid mixture, it cannot be determined whether the observed effects are specific to WPI or reflect a more general amino acid- or protein-driven response. To further distinguish amino acid-mediated effects from potential WPI-specific responses, an amino acid-matched formulation lacking protein and peptide fractions was included as a comparator condition. Additional experiments (Appendix A) using this AA-control Mix indicated that the effects observed following digested WPI treatment were not fully attributable to free amino acid availability alone. While the amino acid mixture partially improved ATP production and protein synthesis, digested WPI induced significantly greater responses, suggesting a potential contribution of digestion-derived bioactive peptides or other peptide fractions. Further peptidomic analyses will be required to identify the specific molecular mediators involved. Therefore, more comprehensive studies are needed to confirm the physiological significance of these observations, including investigations into the absorption and bioavailability of WPI. Overall, the present findings should be interpreted within the context of a simplified pharmacological ExM in vitro model, and all conclusions should be considered exploratory and not directly translatable to physiological exercise adaptation or post-exercise responses in vivo.

## 5. Conclusions

The current in vitro study suggests that WPI influences cellular responses to pharmacological metabolic stress in C2C12-differentiated cells. WPI treatment was associated with a reduction in oxidative and inflammatory signals, including decreases in ROS production and pro-inflammatory cytokines, as well as modulation of stress-related signalling pathways such as p38 MAPK.

In parallel, WPI treatment was associated with modulation of bioenergetic parameters, including ATP production and glycogen levels, as well as modulation of Ca^2+^/Mg^2+^ fluxes. These effects were more consistently observed under the Post-ExM condition at 24 h, suggesting a time-dependent cellular response within this experimental context.

Overall, these findings suggest a possible role for WPI in influencing cellular adaptation to metabolic stress. These results should not be directly extrapolated to physiological exercise recovery or nutritional interventions in humans. Given the limitations of this simplified in vitro ExM model, the present results should be considered preliminary and require further studies to establish their physiological and translational relevance.

## Data Availability

The data presented in this study are available on request from the corresponding author (the Laboratory of Physiology carefully stores raw data to ensure permanent retention under a secure system).

## Abbreviations

The following abbreviations are used in this manuscript:

AAs	Amino acids
AICAR	5-Aminoimidazole-4-carboxamide ribonucleoside
AMPK	AMP-activated protein kinase
ATCC	American Type Culture Collection
ATP	Adenosine triphosphate
ATR	Attenuated total reflectance
BCAAs	Branched-chain amino acids
Ca^2+^	Calcium ion
DMEM	Dulbecco’s Modified Eagle’s Medium
ELISA	Enzyme-linked immunosorbent assay
ERK	Extracellular signal-regulated kinase
ExM	Exercise–Mimetic Mix
FBS	Fetal bovine serum
FNDC5	Fibronectin type III domain-containing protein 5
IL	Interleukin
IL-6	Interleukin 6
IL-17	Interleukin 17
MAPK	Mitogen-activated protein kinase
Mg^2+^	Magnesium ion
mTOR	Mechanistic target of rapamycin
MTT	3-(4,5-dimethylthiazol-2-yl)-2,5-diphenyltetrazolium bromide
OPP	O-propargyl-puromycin
PI3K/Akt	Phosphoinositide 3-kinase/Protein kinase B
Pre-ExM	Pre-exposure treatment protocol
Post-ExM	Post-exposure treatment protocol
PVDF	Polyvinylidene fluoride
ROS	Reactive oxygen species
SD	Standard deviation
SDS-PAGE	Sodium dodecyl sulfate-polyacrylamide gel electrophoresis
TNF-α	Tumour necrosis factor alpha
WPI	Whey protein isolate

## Appendix A

### INFOGEST In Vitro Digestion of WPI

The gastrointestinal digestion of WPI was simulated using the standardised INFOGEST static in vitro protocol [85]. The procedure consisted of three sequential phases:

1. Oral Phase. WPI samples were mixed with simulated salivary fluid containing α-amylase and incubated at 37 °C for 2 min to mimic oral processing.
2. Gastric Phase. The mixture was combined with simulated gastric fluid containing pepsin, adjusted to pH 3.0, and incubated at 37 °C for 2 h under gentle agitation to simulate stomach digestion.
3. Intestinal Phase. Gastric chyme was then mixed with simulated intestinal fluid containing pancreatin and bile salts, adjusted to pH 7.0, and incubated at 37 °C for 2 h to reproduce small intestinal digestion.

After digestion, samples were immediately neutralised, aliquoted, and either directly analysed or applied to C2C12-differentiated cells to study metabolic and structural effects. This two-step approach (in vitro digestion followed by cellular incubation) allowed the assessment of WPI structural changes and bioactivity under conditions mimicking oral intake and muscle cell metabolism.
